# The enzyme activity of mitochondrial trifunctional protein is not altered by lysine acetylation or lysine succinylation

**DOI:** 10.1371/journal.pone.0256619

**Published:** 2021-10-13

**Authors:** Yuxun Zhang, Eric Goetzman

**Affiliations:** Division of Genetic and Genomic Medicine, Department of Pediatrics, University of Pittsburgh School of Medicine, Pittsburgh, Pennsylvania, United States of America; University of Waterloo, CANADA

## Abstract

Mitochondrial trifunctional protein (TFP) is a membrane-associated heterotetramer that catalyzes three of the four reactions needed to chain-shorten long-chain fatty acids inside the mitochondria. TFP is known to be heavily modified by acetyllysine and succinyllysine post-translational modifications (PTMs), many of which are targeted for reversal by the mitochondrial sirtuin deacylases SIRT3 and SIRT5. However, the functional significance of these PTMs is not clear, with some reports showing TFP gain-of-function and some showing loss-of-function upon increased acylation. Here, we mapped the known SIRT3/SIRT5-targeted lysine residues onto the recently solved TFP crystal structure which revealed that many of the target sites are involved in substrate channeling within the TFPα subunit. To test the effects of acylation on substate channeling through TFPα, we enzymatically synthesized the physiological long-chain substrate (2E)-hexadecenoyl-CoA. Assaying TFP in SIRT3 and SIRT5 knockout mouse liver and heart mitochondria with (2E)-hexadecenoyl-CoA revealed no change in enzyme activity. Finally, we investigated the effects of lysine acylation on TFP membrane binding *in vitro*. Acylation did not alter recombinant TFP binding to cardiolipin-containing liposomes. However, the presence of liposomes strongly abrogated the acylation reaction between succinyl-CoA and TFP lysine residues. Thus, TFP in the membrane-bound state may be protected against lysine acylation.

## Introduction

Mitochondrial fatty acid β-oxidation (FAO) is the process by which fatty acids are chain-shortened to produce energy in the mitochondria. FAO is a critical bioenergetic pathway in several tissues, most notably the heart, muscle, and liver [1]. The importance of FAO in these tissues is evidenced by the severity of symptoms in patients suffering from inborn errors of mitochondrial FAO, particularly those with defects in the long-chain FAO enzymes carnitine palmitoyltransferase-2 (CPT2), very long-chain acyl-CoA dehydrogenase (VLCAD), and mitochondrial trifunctional protein (TFP) [1, 2]. Despite the prevalence of newborn screening to identify patients with these disorders, there is no effective therapy beyond dietary management. Quality of life issues and mortality remain a concern.

Understanding the factors that modulate mitochondrial FAO enzyme function and pathway flux may lead to the development of new therapies for inborn errors of FAO as well as more common diseases involving dysfunctional FAO, such as obesity, diabetes, and cancer. To that end, post-translational modifications (PTMs) have emerged as candidates for modulating the activity and function of long-chain FAO enzymes. For example, S-nitrosylation improves the catalytic efficiency of VLCAD [3, 4]. On the other hand, lysine acetylation or succinylation of the membrane-binding domain blocks membrane binding and causes VLCAD mis-localization away from the inner mitochondrial membrane [5]. The effect of PTMs on the other key long-chain enzymes CPT2 and TFP, however, have not been as well delineated.

TFP is an oligomer of two α subunits (encoded by *Hadha* gene) and two β subunits (encoded by *Hadhb* gene). After VLCAD introduces a double-bond between carbons 2 and 3 of a long-chain acyl-CoA substrate, TFPα hydrates the double-bond (enoyl-CoA hydratase activity) and then dehydrogenates the resulting 3-hydroxyacyl-CoA in an NAD^+^-dependent manner, producing a 3-ketoacyl-CoA and NADH. Then, TFPβ, using free CoA in the ketothiolase reaction, cleaves the 3-ketoacyl-CoA to produce acetyl-CoA and an acyl-CoA that is two carbons shorter. TFP is highly prone to PTMs. According to the PTM compendium maintained at www.phosphosite.org, more than 170 PTMs have been identified on TFP, including dozens of lysine acetylation, succinylation, glutarylation, and ubiquitination sites. TFP is one of the most heavily acetylated and succinylated enzymes in the mitochondria [6, 7]. Because these PTMs are thought to occur non-enzymatically in the mitochondria [8, 9], the high degree of lysine acylation on TFP may reflect its exposure to acyl-CoA metabolites in the mitochondrial matrix.

In mitochondria, lysine acetylation and succinylation are reversed by the sirtuin deacylase enzymes sirtuin-3 (Sirt3) and sirtuin-5 (Sirt5), respectively. Sirt3KO and Sirt5KO mice demonstrate hyperacetylation and hypersuccinylation of TFP. The functional ramifications of TFP hyperacylation, however, remain unclear due to conflicting data in the literature. In mouse liver and muscle, increased acetylation of TFP has been associated with lower enzyme activity [10, 11], but in heart acetylation purportedly improves TFP activity [12]. Acetylation increases TFP protein stability in the liver but reduces stability in pancreatic islets [13, 14]. In Sirt5KO liver, increased succinylation had no effect on TFP activity [15], while in Sirt5KO heart, both increased and decreased TFP activities have been reported [16, 17]. However, all of these studies except Goetzman et al. [15] and Sadhukhan et al. [17] utilized a short-chain 3-ketoacyl-CoA (acetoacetyl-CoA, C_4_) in their activity assays, which is the preferred substrate of the short/medium-chain hydroxy-acyl-CoA dehydrogenase (SCHAD) enzyme rather than TFP [18]. Here, we sought to resolve this issue by using physiological long-chain substrates to more specifically assay TFP in Sirt3KO and Sirt5KO mouse liver and heart.

## Materials and methods

### Animal experimentation

All animal studies were approved by the University of Pittsburgh Institutional Animal Care and Use Committee (IACUC) and conducted in accordance with the guidelines and regulations set forth in the Animal Welfare Act (AWA) and PHS Policy on Humane Care and Use of Laboratory Animals. Sirt3KO mice on a 129S1/SvImJ strain background were obtained from Jackson Laboratories (Bar Harbor, ME). Sirt5KO mice, also from Jackson Laboratories but on a mixed background, were backcrossed onto the 129S1/SvImJ strain. 129S1/SvImJ mice were used as wild-type controls. All experimental animals were males ages 10–14 weeks and were fasted for 16 hr prior to tissue collection (N = 5).

### Analysis of TFP acetylation and succinylation sites

Sirt3 and Sirt5-targeted lysine residues on TFP were defined as those residues exhibiting significantly increased (P<0.05) acetylation or succinylation, respectively, in the previously published mass spectrometry datasets of Hirschey et al. [7] (comparing wildtype to Sirt3KO liver) and Rardin et al. [6] (comparing wildtype to Sirt5KO liver). Each sirtuin-targeted site was ascribed to its corresponding TFP catalytic domain: 1) Enoyl-CoA hydratase (ECH) domain, encompassing TFPα residues 37–333; 2) long-chain hydroxyacyl-CoA dehydrogenase (LCHAD) domain, encompassing TFPα residues 334–763; or 3) the ketothiolase (KT) domain, encoded by the TFPβ subunit. Sirtuin-targeted lysines involved in substrate channeling within the TFP holoenzyme were taken from the analysis of the TFP crystal structure presented by Xia et al. [19].

### Enzyme activity assays

SCHAD activity was assayed in reverse following the oxidation of NADH to NAD+ at 340 nm in the presence of 25 μM acetoacetyl-CoA (Sigma, St. Louis, MO) [20]. The LCHAD activity of TFP was assayed in reverse following the oxidation of NADH to NAD+ at 340 nm in the presence of 25 μM 3-ketopalmitoyl-CoA (hereafter keto-C_16_-CoA, Toronto Research Chemicals, Toronto, ON) [15]. Combined enoyl-CoA hydratase (ECH) and LCHAD activities were assayed in the forward direction using 25 μM (2E)-hexadecenoyl-CoA (hereafter enoyl-C_16_-CoA) as substrate. Because enoyl-C_16_-CoA is not commercially available, we used recombinant human acyl-CoA oxidase-1 (ACOX1), expressed in *E coli* and purified as previously described [21], to convert palmitoyl (C_16_)-CoA (Sigma, St. Louis, MO) into enoyl-C_16_-CoA. To determine whether substrate conversion by ACOX1 was complete, we tested the starting C_16_-CoA material and the end product as substrates for VLCAD, a C_16_-CoA dehydrogenase enzyme, in the electron transferring-flavoprotein fluorescence reduction assay [22]. VLCAD specific activity with the starting material was 578 mU/mg, and with the ACOX1 reaction product activity was undetectable, indicating complete conversion. In addition to the above substrates, recombinant human TFP was also assayed with 3-ketodecanoyl-CoA (Sigma, St. Louis, MO). All TFP and SCHAD activity assays were conducted in a reaction buffer containing 100 mM KPO_4_, 50 mM MOPS, 0.1 mM DTT, 0.1% Triton X-100, and 0.15 mM NADH, pH 6.2. The conversion of either NADH to NAD^+^ (reverse assays) or NAD^+^ to NADH (forward assays) was followed in a plate reader at 340 nm. Assays of recombinant TFP contained 1 μg of His-tagged human TFP protein, expressed in *E Coli* using vectors provided by Dr. Jung-ja Kim and purified following her published protocol [19].

Assays of mouse heart and liver used 20 μg of mitochondrial protein. Liver mitochondria were isolated in 225 mM mannitol, 75 mM sucrose, 0.2 mM EDTA, pH7.4. Fatty acid-free BSA (2.5 mg/ml) was added during homogenization and differential centrifugation. After two slow-speed clearing centrifugations (100 × g and 600 × g for 10 min each), the supernatant was centrifuged at 3000 ×g for 10 min. After one wash to remove BSA, the resulting mitochondrial pellets were resuspended in enzyme activity assay buffer. Heart mitochondria were isolated in 300 mM sucrose, 10 mM Na-HEPES, 0.2 mM EDTA, pH7.2. Fatty acid-free BSA (1 mg/ml) was added during homogenization and centrifugation. Two slow-speed clearing centrifugations (100 × g and 600 × g for 10 min each) were performed before pelleting mitochondria at 8000 × g for 15 min. The pellet was washed once and then resuspended in enzyme activity assay buffer.

### Immunoblotting of mouse tissues

Aliquots of total liver or heart mitochondrial protein were electrophoresed on Criterion SDS polyacrylamide gels (BioRad, Hercules, CA) and transferred to nitrocellulose membranes. Primary antibodies used for immunoblotting were: anti-TFPα (Abcam, 1:1000), TFPβ (Santa Cruz, 1:250), anti-SCHAD (Abcam, 1:2000), anti-acetyllysine (Cell Signaling, 1:1000), anti-succinyllysine (PTM Biolabs, 1:1000) and anti-heat shock protein-60 (Hsp60, Cell Signaling, 1:2000) as a mitochondrial marker for loading control. After incubation with HRP-conjugated secondary antibodies (1:5000) blots were visualized with chemiluminescence. The blots were scanned and subjected to densitometric analysis using ImageJ software, normalized to either Hsp60 or to Ponceau S membrane staining.

### Membrane binding studies

Liposomes were prepared from a 3:1 ratio of cardiolipin and dimyristoyl-sn-glycero-3-phosphocholine (DMPC). Lipids were dried by speed vacuum, reconstituted in chloroform, dried a second time, and then dissolved in 25 ul of binding buffer (150 mM KCl, 25 mM Tris-HCl, 1 mM DTT, 0.5 mM EDTA, final pH 7.5). The lipids were incubated at 52°C for 5 hours followed by 1 hr at 37°C and 15 min at room temperature. Aliquots of human recombinant TFP (100 ng) were added and binding done at 37°C for 1 hr. Lipid-bound TFP was separated from non-bound TFP by centrifugation at 42000 ×g at 20°C for 2 hr. The supernatant was carefully removed to new tubes. The pellet and supernatant fractions were then resolved by SDS-PAGE and immunoblotted for TFP. The effect of lysine acylation on TFP cardiolipin binding was determined by incubating recombinant TFP with either succinyl-CoA (0.5 mM) or 50 μM sulfo-NHS-acetate for 30 min at 37°C to induce acylation as described [5, 9]. Non-acylated control samples of TFP were mock-treated and incubated for 30 min at 37°C. Then the proteins were dialyzed to remove excess acylating agents and bound to prepared lipids as described above. In a variation of this experiment, the acylating agents were added simultaneously with the TFP protein to the liposomes (condition #3 in Fig 5E).

## Results

### Sirt3 and Sirt5 target multiple lysine residues involved in substrate channeling within the TFPα subunit

To gain insight into potential mechanisms by which reversible lysine acylation may regulate TFP function, we reanalyzed previously published site-level acylomics datasets generated by mass spectrometry profiling of Sirt3KO and Sirt5KO liver mitochondria. Sirt3 and Sirt5 target sites were defined as lysine residues showing a statistically significant increase (P<0.05) in acetylation or succinylation in Sirt3KO or Sirt5KO liver, respectively. Target sites on TFPα were further subdivided into those occurring in the 2-enoyl-CoA hydratase (ECH) domain, defined as residues 33 to 333, and those occurring in the long-chain 3-hydroxyacyl-CoA dehydrogenase domain (LCHAD), defined as residues 334 to 763. The TFPβ subunit contains only 3-ketothiolase (KT) enzymatic activity.

**Fig 1:**
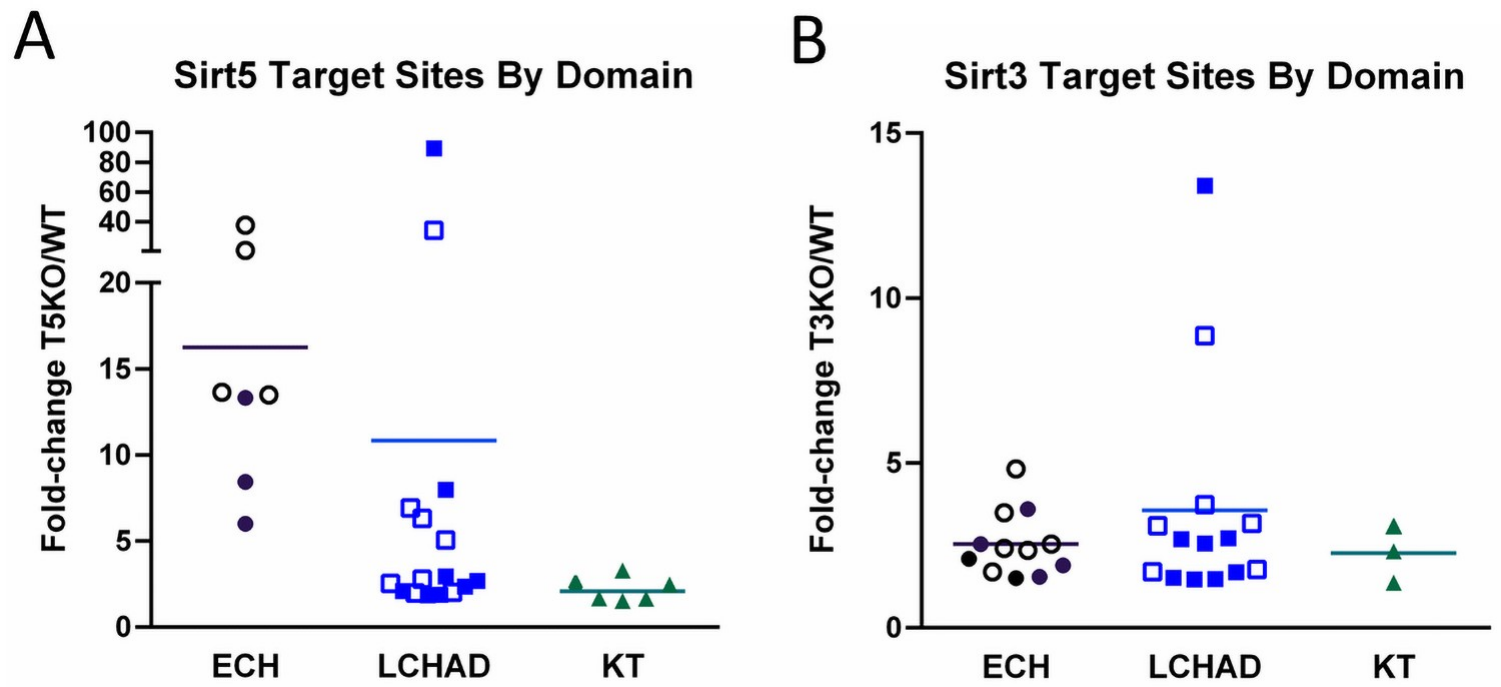
Distribution of known Sirt5 and Sirt3 target sites across the three enzymatic domains of TFP. A) Sirt5 target sites taken from Rardin et al. [6], categorized by location to either the enoyl-CoA hydratase (ECH) of TFPα, the long-chain 3-hydroxyacyl-CoA dehydrogenase (LCHAD) domain of TFPα, or the ketothiolase domain (KT) encoded by TFPβ. B) Sirt3 target sites taken from Hirschey et al. [7] and categorized by TFP enzymatic domain. In both panels the horizontal lines represent the mean fold-change (KO/WT) and open symbols represent sites important for substrate channeling within the TFP complex, determined by the recently published TFP crystal structure [19].

The degree of hyper-succinylation upon Sirt5 deletion was much more dynamic on the ECH and LCHAD domains compared to the KT domain (Fig 1A). There were seven Sirt5 target sites in the ECH domain and 16 in the LCHAD domain with mean fold-changes in succinylation of 16.2 and 10.8, respectively. In contrast, the mean fold-change in succinylation for the six Sirt5 target sites in the KT domain (TFPβ) was only 2.2. In Sirt3KO liver, there were 12 significantly hyperacetylated sites in the ECH domain (mean fold-change 2.5), 14 sites in the LCHAD domain (mean fold-chage 3.6), and only three sites in the KT domain (mean fold-change 2.3) (Fig 1B). Together these results suggested that the TFPα subunit is subject to more reversible lysine acylation than the TFPβ subunit.

Finally, we used the recently solved three-dimensional crystal structure of TFP [19] to gain further insight into possible mechanisms by which lysine acylation may affect TFP function. Interestingly, about half of the Sirt5 target sites and half of the Sirt3 target sites are predicted to play a role in substrate channeling either between the ECH and LCHAD domains within the TFPα subunit or between TFPα and TFPβ [19]. These target sites are indicated with open symbols in Fig 1A and 1B.

### Optimizing activity assays of LCHAD and ECH-LCHAD channeling using 3-ketohexadecanoyl-CoA and 2-enoyl-hexadecanoyl-CoA as substrates

**Fig 2:**
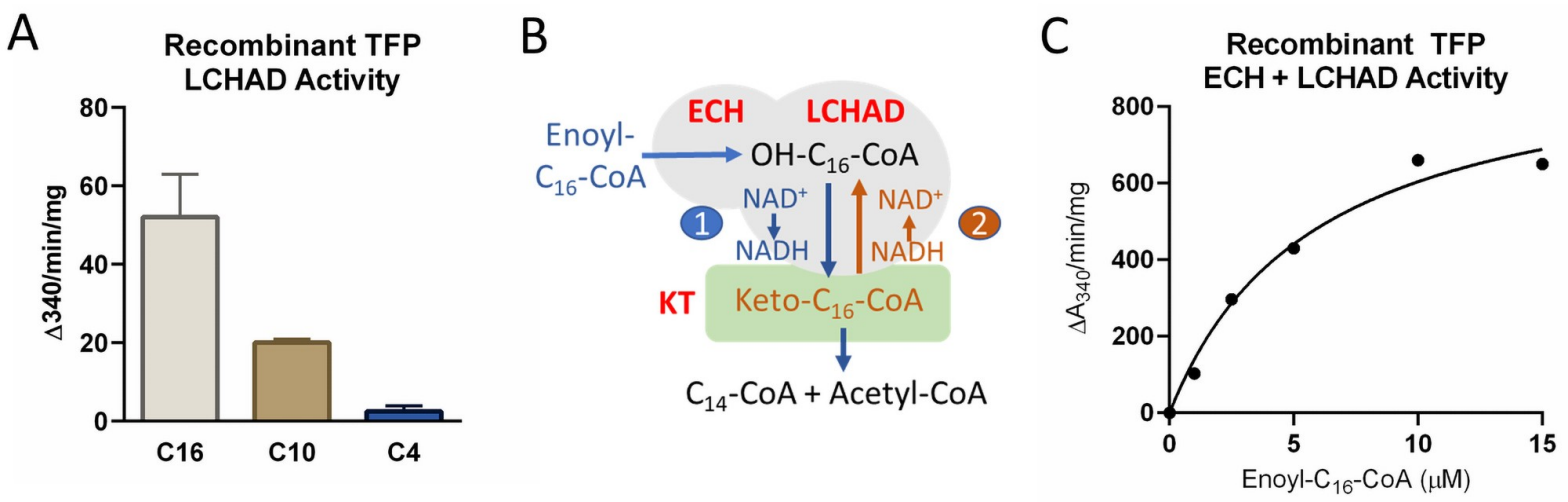
Optimization of activity assays for TFP. 16, 10 and four-carbon 3-keto-acyl-CoA substrates have all been used in the literature to assay the long-chain 3-hydroxyacyl-CoA dehydrogenase (LCHAD) activity of TFP in the reverse direction. Panel A) shows the relative activity of recombinant TFP with all three of these substrates in the reverse direction with a saturating substrate concentration (25 μM). B) Schematic outlining the two strategies that were used to assay TFPα activity in Figs 3 and 5. Strategy #1 (blue font) uses enoyl-C_16_-CoA, synthesized enzymatically from C_16_-CoA, to assay the combined enoyl-CoA hydratase (ECH) and LCHAD activities by following the reduction of NAD^+^ to NADH (forward direction). This assay requires substrate channeling between the two TFPα active sites. Strategy #2 (brown font) is to measure the isolated LCHAD activity in reverse using keto-C_16_-CoA and following conversion of NADH to NAD^+^. C) Demonstration that recombinant TFP responds to the enzymatically synthesized enoyl-C_16_-CoA substrate in a dose-dependent manner when assayed with strategy #1.

Several previous investigations have probed the effects of lysine acylation on TFP function with conflicting results. These studies measured TFP’s LCHAD activity in reverse. Most used acetoacetyl-CoA, a four-carbon 3-ketoacyl-CoA, as substrate, with the exception of Sadhukhan et al. [17] who used a 10-carbon 3-ketoacyl-CoA as substrate and Goetzman et al. [15] who used both the 10 and 16-carbon 3-ketoacyl-CoAs. To objectively test the substrate specificity of the reverse LCHAD assay, we assayed recombinant human TFP in reverse with acetoacetyl-CoA (C_4_), 3-ketodecanoyl-CoA (C_10_), and 3-ketopalmitoyl-CoA (C_16_). Compared to C_16_, the relative LCHAD activities against the C_10_ and C_4_ ketoacyl-CoA species were 40% and 5%, respectively (Fig 2A). For all subsequent reverse measurements of LCHAD activity the keto-C_16_-CoA substrate was used.

Next, given the high number of Sirt3 and Sirt5 target sites among lysine residues thought to be critical for substrate channeling within the TFPα subunit, we sought to assay the combined ECH/LCHAD activities of TFPα using the ECH substrate enoyl-C_16_-CoA. Because enoyl-C_16_-CoA is not commercially available, we synthesized this substrate enzymatically in-house using purified recombinant human acyl-CoA oxidase-1 (ACOX1) and palmitoyl-CoA as the starting material. The ACOX1-generated enoyl-C_16_-CoA was an excellent substrate for recombinant TFP using NAD+ reduction to NADH as the readout of the combined forward activities of ECH and LCHAD (Fig 2B and 2C).

### Effects of Sirt3 and Sirt5 ablation on SCHAD, LCHAD, and ECH-LCHAD enzyme activities in mice

Previous studies attempting to test TFP function in Sirt3/Sirt5 knockout mouse tissue lysates used substrates more suited to the measurement of SCHAD. Here, we used acetoacetyl-CoA to assay SCHAD activity in reverse, 3-keto-C_16_-CoA to measure isolated LCHAD activity in reverse (following NADH oxidation to NAD^+^, assay scheme #1, Fig 2B), and 2-enoyl-C_16_-CoA to measure combined ECH-LCHAD activity in the natural, forward direction (assay scheme #2, Fig 2B). To eliminate potential artifacts caused by the two peroxisomal multifunctional enzymes, which can also utilize the 16-carbon substrates, mitochondria were isolated by differential centrifugation from wild-type, Sirt3KO, and Sirt5KO mouse livers and hearts.

**Fig 3:**
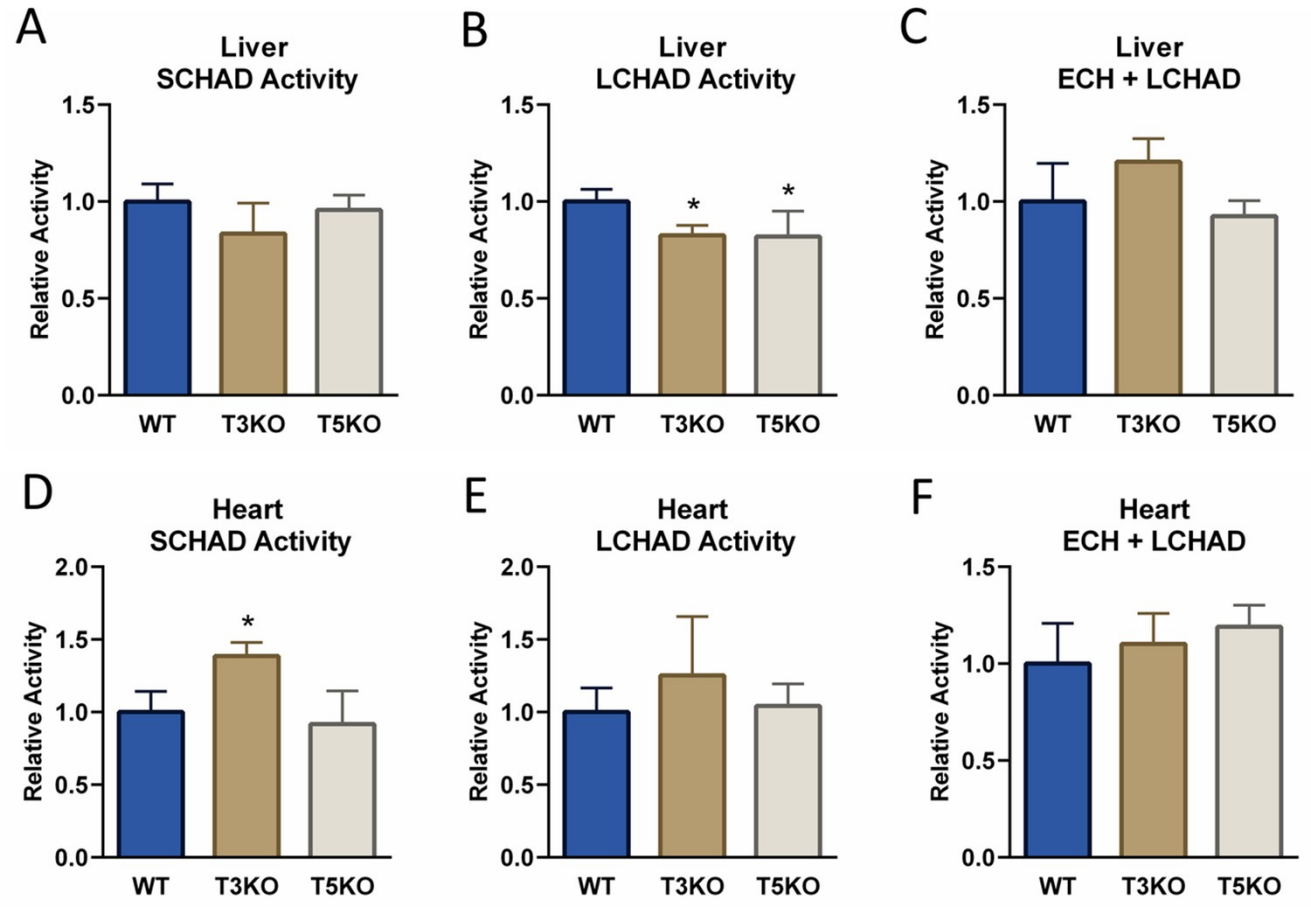
SCHAD and TFP enzyme activities in Sirt3KO and Sirt5KO mouse liver and heart mitochondria. Mitochondria were isolated from N = 5 wild-type (WT), Sirt3KO (T3KO), and Sirt5KO (T5KO) mouse livers and hearts and assayed in the reverse direction for SCHAD activity with acetoacetyl-CoA (panels A and D), in the reverse direction for isolated LCHAD activity with keto-C_16_-CoA (panels B and E), and in the forward direction with channeling of enoyl-C_16_-CoA through the combined ECH and LCHAD activities of TFPα (panels C and F). *P<0.05 versus wild-type. All bars represent means and standard deviations.

**Fig 4:**
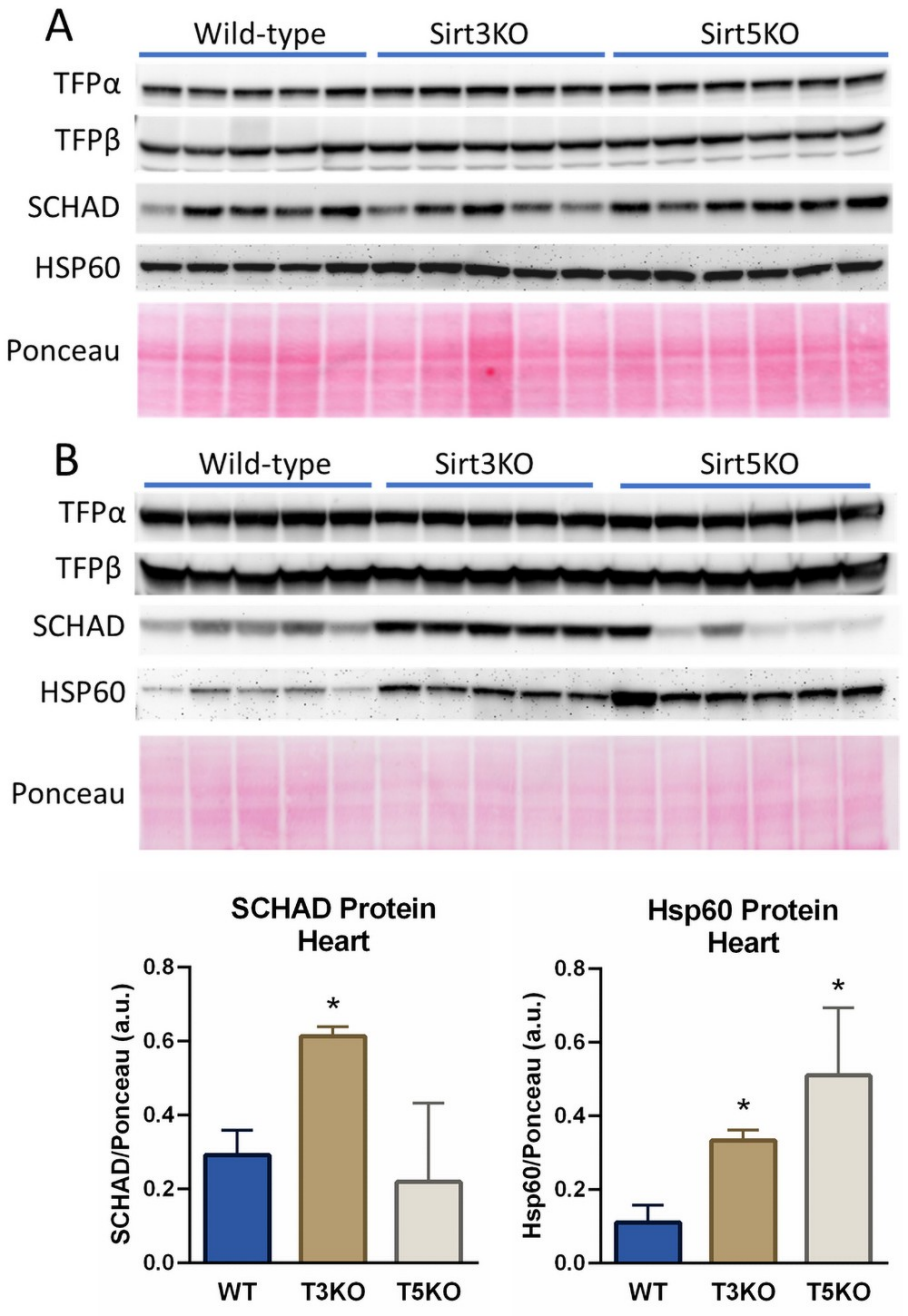
Immunoblotting of SCHAD and TFP in isolated Sirt3KO and Sirt5KO mouse liver and heart mitochondria. Mitochondria isolated from N = 5 wild-type (WT), N = 5 Sirt3KO (T3KO), and N = 6 Sirt5KO (T5KO) mouse livers (A) and hearts (B) were immunoblotted for both subunits of TFP, SCHAD, and mitochondrial heat shock protein-60 (HSP60) as a mitochondrial loading control. Densitometry was used to normalize expression of SCHAD and TFP to both HSP60 and ponceau membrane staining. No statistically significant differences were observed in liver (densitometry results not shown), while in heart differential expression of SCHAD and HSP60 was observed, with densitometry results shown in bar graphs below panel B. Bars represent means and standard deviations, with *P<0.05 versus wild-type control.

Liver mitochondrial SCHAD activity was not significantly altered by deletion of either Sirt3 or Sirt5 (Fig 3A). In contrast, isolated LCHAD activity measured with the reverse assay using keto-C_16_-CoA was significantly reduced in liver from both knockout strains (~20% reduction, Fig 3B). However, the forward combined ECH/LCHAD assay produced a conflicting result to the reverse LCHAD assay, as there was no significant change in total TFPα activity for liver mitochondria from either knockout strain (Fig 3C). Western-blotting followed by densitometry (densitometry results not shown) confirmed that the expression levels of SCHAD and TFP were not altered in either Sirt3KO or Sirt5KO liver mitochondrial isolates, with the mitochondrial chaperone Hsp60 used as a loading control (Fig 4A).

In heart, increased SCHAD activity was observed in Sirt3KO mitochondria but not Sirt5KO mitochondria (Fig 3D), which was coincident with significantly increased expression of the SCHAD protein (Fig 4B blot and bar graph). No effects were seen on TFPα activity in Sirt3KO and Sirt5KO heart mitochondria with either the reverse LCHAD assay or the forward ECH/LCHAD assay (Fig 3E and 3F). TFP antigen levels also were not changed as indicated by western blotting and densitometry (Fig 4B; densitometry not shown). Interestingly, expression of the mitochondrial chaperone Hsp60 was significantly increased in both Sirt3KO and Sirt5KO cardiac mitochondria, suggestive of misfolded protein stress in these hearts. As a result of this, ponceau staining was used as a loading control during densitometric analysis for the blots presented in Fig 4B.

### Lysine succinylation does not disrupt TFP membrane binding

**Fig 5:**
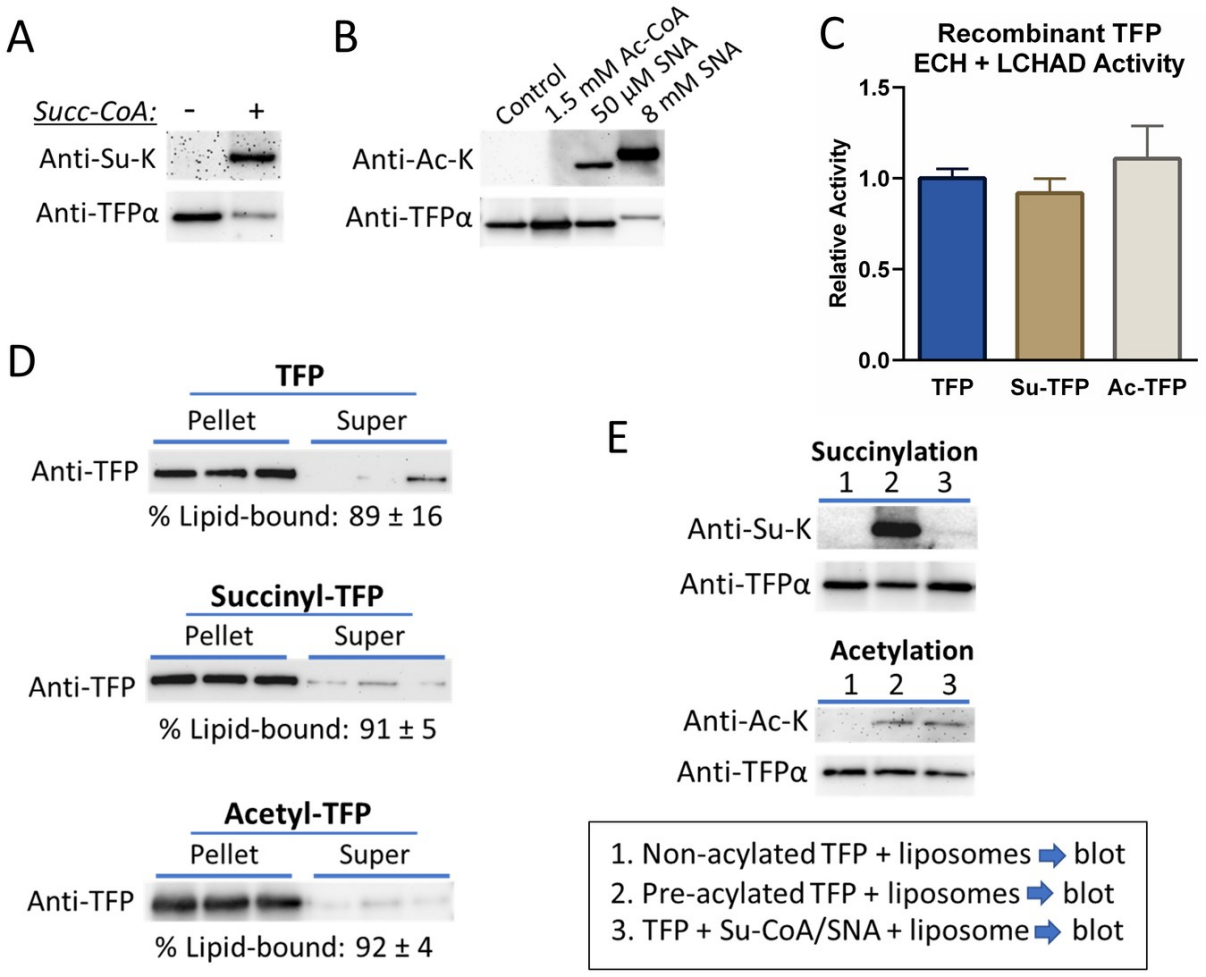
Acylation of recombinant TFP does not alter activity or membrane binding. A) Recombinant human TFP treated with 0.5 mM succinyl-CoA for 30 minutes exhibits lysine succinylation as shown by anti-succinyllysine immunoblotting of 100 ng of TFP protein. B) Optimization of *in vitro* acetylation conditions for TFP. Shown is an anti-acetyllysine immunoblot of TFP (100 ng per lane) treated with acetyl-CoA (1.5 mM) versus the acetylating agent sulfo-NHS-acetate (SNA) at either 50 μM or 8 mM. SNA at 50 μM was chosen for subsequent experiments. C) Recombinant human TFPα enzyme activities in the forward direction with 25 μM 2-enoyl-C_16_-CoA after succinylation and acetylation as described in panels A and B. Bars represent means and standard deviations of three biological replicates each assayed 3–4 times (10 total activity measurements), normalized to untreated control TFP. D) Triplicate samples of untreated control recombinant human TFP, succinylated TFP, and SNA-acetylated (50 μM) TFP were incubated with cardiolipin-containing liposomes for 30 minutes and then ultracentrifuged. The lipid pellet and supernatant fractions were then blotted with anti-TFPα antibody. Densitometry was used to estimate the % of lipid-bound TFP in the pellet fraction (shown below each immunoblot), which did not change with lysine acylation. The entire experiment was repeated with similar results. E) The effect of cardiolipin liposomes on the acylation of recombinant TFP was tested by either pre-acylating the protein for 30 min (condition #2) or mixing protein, acylating agent, and liposomes all at once for 30 min prior to blotting (condition #3). The entire experiment was repeated a second time with similar results.

We previously showed that lysine acylation of VLCAD, the enzyme just upstream of TFP in the mitochondrial FAO pathway, disrupts binding to cardiolipin, resulting in protein mis-localization away from the inner mitochondrial membrane [5]. Like VLCAD, TFP is a membrane-associated protein known to bind specifically to cardiolipin [23]. To determine if lysine acylation could affect TFP membrane binding, we utilized an *in vitro* protein acylation strategy with recombinant human TFP. Succinyl-CoA is highly reactive acyl-CoA metabolite which chemically acylates proteins both *in vitro* and *in vivo* [8, 9]. Indeed, incubation of recombinant TFP with 0.5 mM succinyl-CoA for 30 minutes produced robust lysine succinylation as observed with anti-succinyllysine immunoblotting (Fig 5A). Acetyl-CoA was reported to be much less efficient as an acetylating agent, which we confirmed by incubating 1.5 mM acetyl-CoA with recombinant TFP followed by anti-acetyllysine immunoblotting (Fig 5B, lane 2). We previously circumvented this problem by acetylating VLCAD and other FAO proteins with 8 mM sulfo-NHS-acetate (SNA) [5, 24]. Here, 8 mM SNA strongly acetylated TFP to the extent that a mass-shift of several kDa was observed (Fig 5B, lane 4). However, TFP acetylated in the manner was unstable and completely inactive (data not shown). Through optimization of SNA dosing, 50 μM SNA was found to robustly acetylate TFP without compromising stability (Fig 5B, lane 3). Neither acetylation of TFP with 50 μM SNA or succinylation with 0.5 mM succinyl-CoA changed the enzymatic activity of recombinant TFP (Fig 5C).

Next, we compared the cardiolipin-binding capacity of succinylated and acetylated recombinant TFP to mock-treated TFP. After incubation of each protein with cardiolipin-containing liposomes, ultracentrifugation was used to separate lipid-bound TFP from non-bound TFP. Under these conditions, about 90% of control TFP, succinylated TFP, and acetylated TFP localized to the liposome pellet (Fig 5D), indicating that, unlike VLCAD, the cardiolipin binding of TFP is not sensitive to lysine acylation. In a final experiment, we observed that the presence of lipids greatly reduces the ability of succinyl-CoA, but not SNA, to react with TFP’s lysine residues. Three conditions were compared: 1) recombinant TFP was mock-treated (non-acylated), dialyzed, and then mixed with cardiolipin-containing liposomes; 2) TFP was pre-acylated with either succinyl-CoA or SNA, dialyzed to remove the acylating agent, and then mixed with liposomes; and 3) TFP protein, acylating agent, and liposomes were all mixed at the same time. Anti-succinyllysine immunoblotting showed that the presence of liposomes almost completely inhibited the reaction of succinyl-CoA with TFP lysine residues, as evidenced by very low TFP succinylation (Fig 5E, left panel, compare lanes 2 and 3). The effect of the acetylating agent SNA, however, was not affected by the presence of liposomes (Fig 5E, right panel, compare lanes 2 and 3).

## Discussion

Over the past decade, several mitochondrial enzymes have been identified that display altered function when acylated on lysine residues, including FAO enzymes, enzymes in the TCA cycle and ketogenesis pathways, and components of the electron transport chain [25, 26]. In general, increased acylation is associated with reduced enzyme stability and reduced activity, with a few notable exceptions. IFor instance, ncreasedacetylation was associated with increase activity of the FAO enzymes long-chain acyl-CoA dehydrogenase (LCAD) and TFP in mouse heart [12, 27], while another study observed increased TFP activity in Sirt5-/- mouse heart, presumably due to hyper-succinylation [16]. However, this latter finding was directly contradicted by Sadhukhan, et al, who saw decreased TFP activity with increasing succinylation [17]. In the context of inborn errors of metabolism (IEMs), acyl-CoAs accumulate and can alter the lysine acylome inside the mitochondria [28]. This can result in secondary changes to the function of other enzymes and pathways. The best example of this is glutaryl-CoA dehydrogenase deficiency, which leads to accumulation of glutaryl-CoA, causing increased lysine glutarylation via chemical acylation, ultimately leading to reduced function of other mitochondrial proteins such as carbamoyl phosphate synthase-1 (Cps1) in the urea cycle [29]. Understanding the tissue and enzyme-specific consequences of lysine PTMs can shed light on the pathogenesis of IEMs, and further, modulating PTMs via pharmacological targeting of sirtuin activities may be a promising therapeutic avenue [30].

As detailed in the Introduction, we noted conflicting reports regarding the effects of acylation and mitochondrial sirtuins on TFP function. We hypothesized that many of these conflicting results could be due to the use of acetoacetyl-CoA as substrate when measuring TFP activity. Because 3-hydroxyacyl-CoAs have historically not been commercially available, the convention has been to measure SCHAD activity in reverse with its product acetoacetyl-CoA and LCHAD activity with its product keto-C_16_-CoA [20]. However, due to confusion over substrate specificity and to difficulties finding a commercial source for keto-C_16_-CoA, in recent years researchers turned to the use of acetoacetyl-CoA as a substrate for TFP activity. Here, we confirmed that recombinant TFP exhibits near-zero activity with acetoacetyl-CoA, and thus conclude that many previous investigations of TFP were actually measuring SCHAD activity. Complicating matters is the existence of a second short-chain hydroxyacyl-CoA dehydrogenase enzyme encoded by the Hsd17B10 gene. The HSD17B10 enzyme is also active with acetoacetyl-CoA [31], and further, is known to be deacetylated and regulated by Sirt3 in certain tissues [32].

As of 2019, keto-C_16_-CoA is available through Toronto Research Chemicals, Inc (Toronto, ON) and can be used for the measurement of TFP’s LCHAD activity. However, use of this substrate may also be problematic for the following reasons. First, SCHAD is known to exhibit partial activity with keto-C_16_-CoA [20]. Second, and perhaps more important, assaying TFP in the reverse direction may yield misleading results. Enzymes are well known to behave differently in the forward direction versus the reverse [33, 34]. Here, we observed a small but statistically significant loss of LCHAD activity in Sirt3KO and Sirt5KO liver when assayed in the reverse direction with keto-C_16_-CoA, but when the complete TFPα activity was assayed in the physiological forward direction with enoyl-C_16_-CoA this difference between genotypes disappeared. The forward direction requires the ECH domain to convert enoyl-C_16_-CoA to 3-hydroxy-C_16_-CoA and channel it to the LCHAD active site. It is possible that the ECH reaction is rate-limiting in this regard, which would make small losses of LCHAD activity physiologically irrelevant. We propose that the enoyl-C_16_-CoA forward assay more closely models the *in vivo* function of the intact TFP enzyme and is therefore superior to the reverse assay with keto-C_16_-CoA, but recognize the limited applicability of the forward assay given that there is no commercial source for the substrate.

While the data presented here indicate that lysine acetylation and succinylation do not affect TFP enzyme activity, one caveat of our approach is that we did not measure the thiolase activity of TFPβ. Based on the fact that acylation in general is less prevalent on TFPβ than TFPα, and that ablating Sirt3 and Sirt5 does little to change acylation levels (see Fig 1) compared to TFPα, we speculated that TFPβ would not be affected by acylation. We attempted to experimentally address this by assaying TFPβ in the forward direction with the keto-C_16_-CoA substrate as has been described [20], but in our hands this assay was inconsistent, particularly when applied to isolated mouse mitochondria. We therefore cannot rule out the possibility that reversible acylation may affect the thiolase activity of TFPβ or the channeling of intermediates from TFPα to TFPβ. Future studies are needed to optimize an HPLC or mass spectrometry-based assay that would take into account all three TFP activities in concert. Similarly, we also cannot rule out the possibility that acylation alters TFP protein homeostasis, which was not addressed here. It is known that TFP is ubiquitinated on 27 different lysine residues, and 24/27 of these are also subject to acylation (www.phosphosite.org). At present, the role of lysine ubiquitination in regulating individual mitochondrial protein turnover is not understood. The potential exists for significant acylation-ubiquitination crosstalk, in which elevated acylation could compete with ubiquitination at the same lysine residues and perhaps influence protein turnover. Intriguingly, we observed here that the presence of cardiolipin-containing liposomes greatly suppressed the chemical reactivity of TFP lysine residues toward succinyl-CoA (Fig 5E). While our data is limited to *in vitro* studies in an artificial membrane system that may not truly recapitulate the mitochondrial membrane *in vivo*, it is tempting to speculate that highly acylated forms of TFP *in vivo* may represent partially unfolded, compromised protein that dislodged from the membrane and is destined for ubiquitination and degradation. Altered protein homeostasis in general could explain the higher abundance of Hsp60—a known molecular chaperone recruited by unfolded protein stress [35]—observed in Sirt3KO and Sirt5KO hearts (Fig 4B). A further layer of complexity is added by the fact that Hsp60 itself, as well as its chaperonin partner Hsp10, are both functionally affected by lysine acylation [36, 37], which could secondarily affect the stability of many mitochondrial proteins including TFP. Future investigations are required to understand the significance of these phenomena.

## Supporting information

S1 Fig Uncropped western blot images related to Fig 4A. (DOCX)

S2 Fig Uncropped western blot images related to Fig 4B. (DOCX)

S3 Fig Uncropped western blot images related to Fig 5. (DOCX)
