# Supplementary figures and images for: The enzyme activity of mitochondrial trifunctional protein is not altered by lysine acetylation or lysine succinylation
Source: PLoS One. 2021 Oct 13;16(10):e0256619. doi: 10.1371/journal.pone.0256619 (PMC8513871; doi:10.1371/journal.pone.0256619)

**Fig S1**


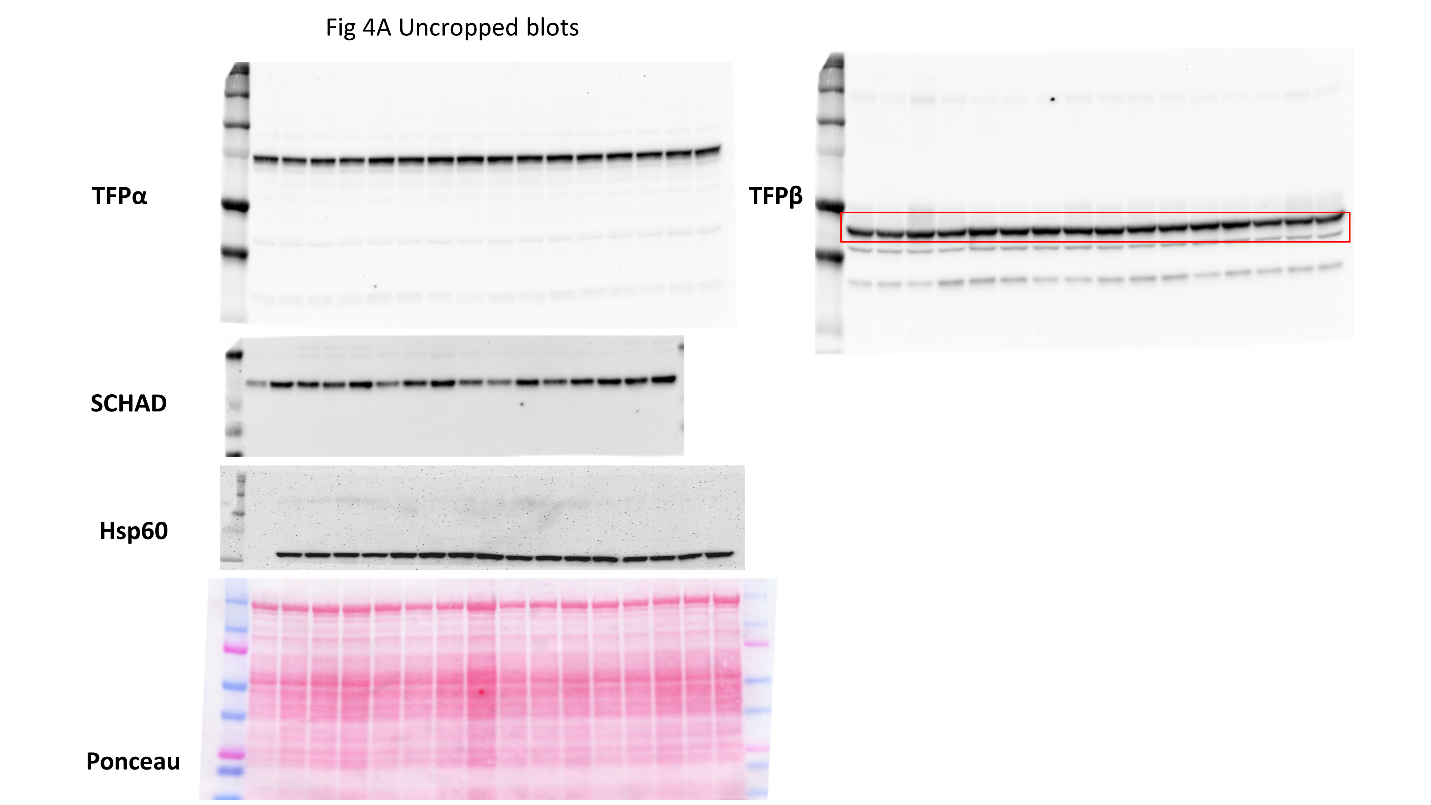

Supplement: S1 Fig — (DOCX) [file pone.0256619.s001.docx]

**Fig S2**

**
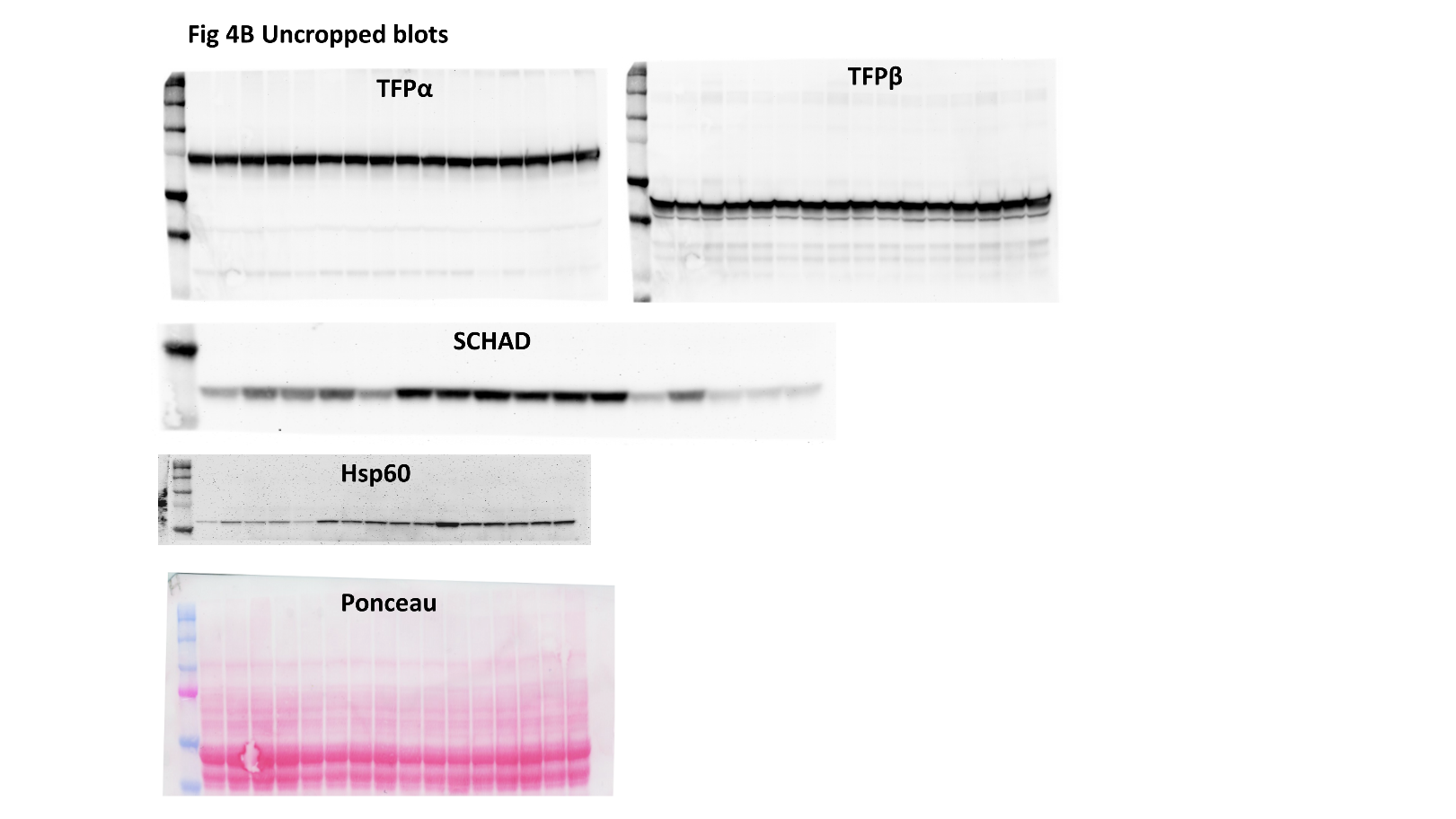
**

Supplement: S2 Fig — (DOCX) [file pone.0256619.s002.docx]

**Fig S3**

**
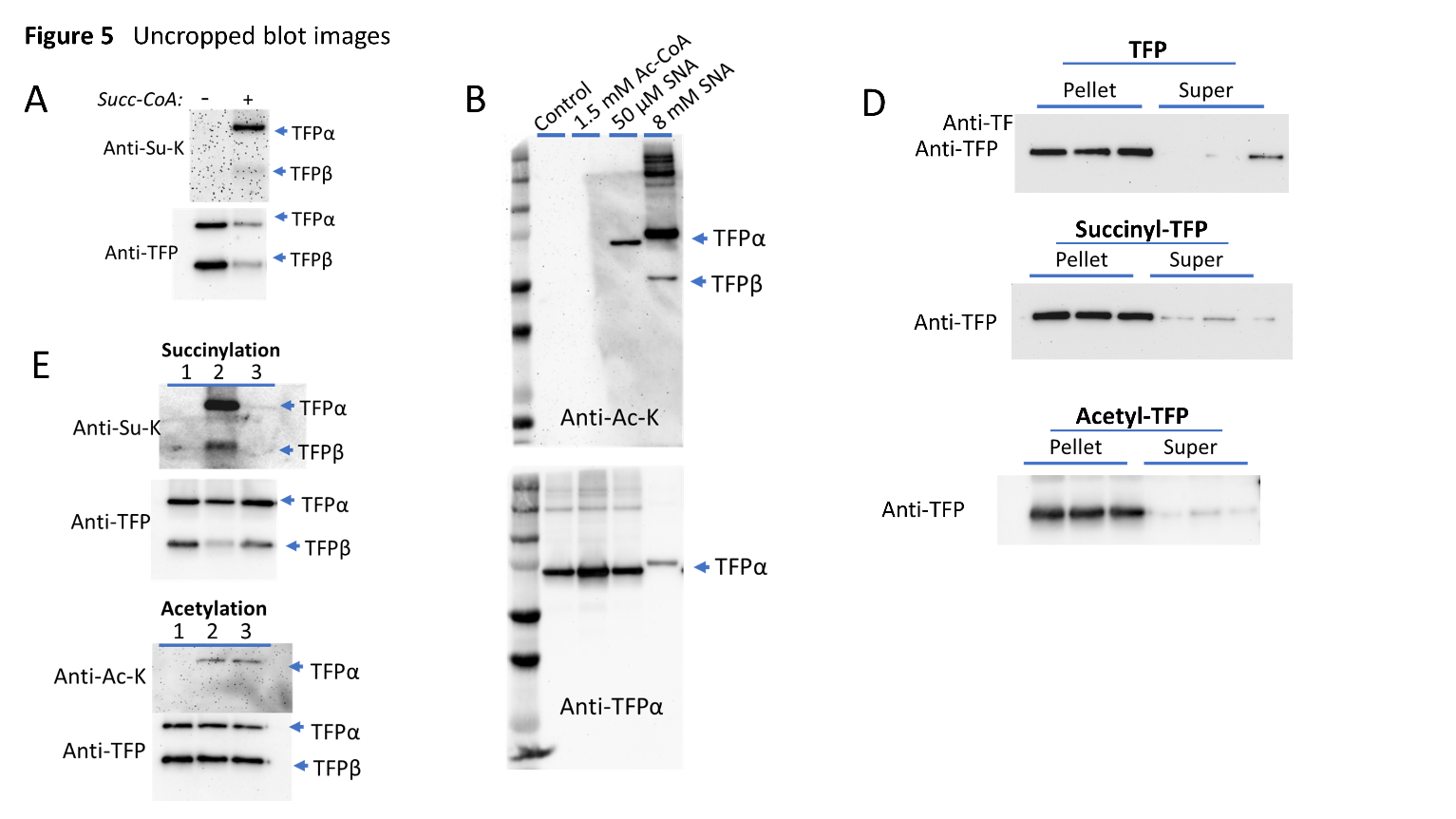
**

Supplement: S3 Fig — (DOCX) [file pone.0256619.s003.docx]
